# Baseline neuropsychological profiles in prion disease predict survival time

**DOI:** 10.1002/acn3.51115

**Published:** 2020-09-09

**Authors:** Saranya E. Sundaram, Adam M. Staffaroni, Nicole C. Walker, Kaitlin B. Casaletto, Megan Casey, Aili Golubjatnikov, Stacy Metcalf, Kelly O’Leary, Katherine Wong, Kendra Benisano, Sven Forner, Marta Gonzalez Catalan, Isabel E. Allen, Howard J. Rosen, Joel H. Kramer, Michael D. Geschwind

**Affiliations:** ^1^ Department of Neurology, Memory and Aging Center Weill Institute for Neurosciences University of California San Francisco California; ^2^ Department of Psychology Palo Alto University Palo Alto California; ^3^ Department of Psychology California School of Professional Psychology Alliant International University San Francisco California; ^4^ Department of Epidemiology and Biostatistics University of California San Francisco California

## Abstract

**Objective:**

Few studies have captured the neuropsychological profile of sporadic Creutzfeldt–Jakob disease (sCJD) with neuropsychological testing, and little is known about cognitive predictors of survival. We characterized baseline neuropsychological performance in sCJD and investigated associations with survival.

**Methods:**

sCJD participants who completed the MMSE (*n* = 118), 61 sCJD of whom also completed a neuropsychological battery at baseline, and 135 age‐matched healthy controls, were included. Composite scores of global cognition, memory, executive functions, visuospatial, and language were derived. Cox proportional hazard models estimated survival time, controlling for age and education. Additional models adjusted for Barthel Index and *PRNP* codon 129 polymorphism.

**Results:**

sCJD participants performed significantly worse than controls on all cognitive tasks and composites with most showing very large effect sizes. The three tests showing the largest group differences were delayed verbal recall (Hedges’*g* = 4.08, *P* < 0.0001), Stroop Inhibition (Hedges’g = 3.14, *P* < 0.0001), and Modified Trails (Hedges’g = 2.94, *P* < 0.0001). Memory (95%) and executive functioning (87%) composites were most commonly impaired. Poorer global (HR = 0.65, *P* < 0.0001), visuospatial (HR = 0.82, *P* < 0.0001), and memory (HR = 0.82, *P* = 0.01) composites predicted shorter survival. Visuospatial cognition remained a significant predictor even after adjusting for all other cognitive composites; each standard deviation decrease in visuospatial cognition was associated with an 18% higher chance of death (HR = 0.82, *P* < 0.003). Global (HR = 0.68, *P* = 0.03) and visuospatial (HR = 0.82, *P* = 0.001) composites remained significant predictors after controlling for Barthel Index and codon 129.

**Interpretation:**

sCJD participants exhibit a broad range of cognitive impairments, with memory and executive functioning deficits in the vast majority. Neuropsychological assessment, particularly of visuospatial abilities, informs prognostication in sCJD.

## Introduction

Human prion diseases are uniformly fatal neurodegenerative diseases that usually present as a rapidly progressive dementia caused by the misfolding of normal prion proteins into abnormally folded prion proteins (prions).^1^ Approximately 85–90% of human prion cases worldwide, and 80–95% of cases in the United States, are sporadic (sCJD).^2^, ^3^, ^4^ The clinical presentations of sCJD are diverse and include cognitive impairment, myoclonus, ataxia, extrapyramidal signs, and other features.^5^ Early diagnosis of sCJD is challenging in the context of heterogeneous and protean clinical manifestations.

In sCJD, cognitive impairment is often the first reported symptom.^6^ Despite this, there is a dearth of research using standardized neuropsychological batteries to characterize cognition in large cohorts of sCJD, due in large part to the rapidly progressive nature and rarity of the condition. Neuropsychological profiling of prion disease has generally been limited to mixed prion cohorts,^7^ case studies,^8^ or other CJD subtypes (e.g., genetic and variant prion disease).^9^, ^10^ One interesting retrospective study tried to capture the neuropsychological profile of a large sCJD cohort, including looking at different disease tertiles, but this study was mostly based on clinical features noted by clinicians or in medical records, and included a wide variety of heterogenous neuropsychological testing that was not administered with any consistency across patients.^11^ In group‐level statistical analyses of prion diseases, impairments are reported in most cognitive domains, with the majority of studies finding impairments in executive functions,^7^, ^9^, ^12^ episodic memory,^9^, ^12^ and language.^7^ When analyzing the individual, however, it is evident that sCJD can present as focal clinical syndromes, such as aphasias^11^, ^13^, ^14^ and visuospatial dysfunction.^7^, ^9^, ^11^

In addition to the need for classifying baseline cognitive profiles, accurate prediction of survival time is needed for clinical care and patient stratification in clinical trials.^15^ Survival rates in sCJD vary, typically lasting around 6 months after initial onset of symptoms, but can range from a few weeks to several years, with 85–90% of participants surviving less than 1 year from onset of first symptoms.^13^, ^16^, ^17^, ^18^ Longer survival time has been associated with demographic and biological factors, including younger age at illness onset,^18^ female sex,^18^ codon 129 heterozygosity,^18^, ^19^, ^20^, ^21^ lower fluid biomarker levels (e.g., plasma and CSF tau),^20^, ^22^, ^23^, ^24^, ^25^ and type 2 prion protein type.^18^, ^26^ The potential role of utilizing cognitive performance to predict survival time remains unknown, however. Most studies have utilized clinical interviews rather than objective neuropsychological testing to determine initial cognitive symptoms. These efforts have revealed that when prion disease first manifests with visuospatial impairments and is characterized by predominant visual symptoms (e.g., poor vision, perceptual disturbances in colors and structures, and optical distortions) throughout the disease course, termed the Heidenhain variant,^27^, ^28^ survival time is often reduced.^28^

The present study aimed to characterize the neuropsychological profile of sCJD and determine whether standardized cognitive testing provides useful information for predicting survival time in this cohort.

## Methods

### Subjects and informed consent

Participants were referred to the University of California, San Francisco (UCSF) Memory and Aging Center from March 2004 to January 2018 and participated in our prion and rapidly progressive dementia (RPD) clinical research program approved by our Institutional Review Board (IRB). All procedures in this study were approved by the UCSF IRB. All participants and/or their study partners provided written informed consent prior to undergoing any study procedures.

Participants considered for this analysis included 248 individuals with probable^29^ (23%) and definite (pathology proven; 77%)^30^ sCJD who underwent a neurologic examination at our center. Of those, 124 subjects who were able to participate in the Mini Mental Status Examination (MMSE)^31^ were considered for this study. Six participants who were placed on life‐extending treatments were excluded from the survival analyses, for a final sample of 118 participants, of whom 77% were pathology‐proven and 23% met probable sCJD criteria.^32^, ^33^ Basic demographics on this cohort are shown in Supplementary Table S1, the *PRNP* codon 129 and prion molecular classification are shown in Table S1. Participants were an average of 60% of the way through their disease course (10.2 months, +/− 7.7 SD). Almost all participants (97%, 114/118) had *PRNP* sequencing to rule out genetic prion disease and determine the codon 129 polymorphism; none had a suggestive family history, and 69% (81/118) had prion typing information available through brain autopsy tissue (National Prion Disease Pathology Surveillance Center (NPDPSC), Cleveland, OH). A subcohort of 61 participants (52%) also completed at least a minimum number of items on comprehensive neuropsychological testing at baseline sufficient to create at least two cognitive composite scores (see Statistical Analyses), which was used in the survival analyses. The remaining 57 subjects (48%) who were able to complete the MMSE but were too impaired at baseline to undergo or to complete a minimum number of tests on this comprehensive neuropsychological battery battery (see Statistical Analyses) were excluded from the survival analyses. This subgroup with MMSE only had no significant difference in demographic parameters from the 61 subjects who were able to do the neuropsychological test battery other than lower MMSE, as expected. Other demographic parameters, except for education, did not show significant differences between these two subgroups (Table 1).

**Table 1:** Baseline demographic and clinical characteristics

	sCJD total sample^1^	Neuropsychological survival analyses^1^	sCJD MMSE‐only sample^1^	Healthy controls^1^	*P‐value* ES^4^	*P‐value* ES^4^	*P‐value* ES^4^
*N*	118	61^2^	57	135			
–	–	–	–	–	sCJD (*n* = 118) and HC	Between sCJD (*n* = 61) and HC	Between sCJD (*n* = 61) and (*n* = 57)
Age at visit (years)	64.5 ± 9.6	65.4 ± 9.4	63.5 ± 9.8	63.1 ± 7.3	*0.19*	*0.07*	*0.31*
	65.2 [57.9, 71.2]	66.3 [58.1, 72.6]	62.9 [57.1, 69.6]	64.0 [59.0, 68.0]	0.17	0.29	0.19
	38.2–88.6	42.7–82.2	38.2–86.6	36.0–81.0			
Education (years)	15.6 ± 2.5	16.0 ± 2.4	13.3 ± 2.1	17.5 ± 1.9	***<0.0001***	***<0.0001***	***0.014***
	16.0 [14.0, 17.5]	16.0 [14.0, 18.0]	12.0 [12.0, 16.0]	18.0 [16.0, 18.0]	**0.91**	**0.73**	**1.14**
	12.0–20.0	12.0–20.0	12.0–16.0	12.0–22.0			
Sex (M/F)	68/50	40/21	28/29	78/57	*0.98* *	*0.30* *	*0.10*
Race (% white)	80%	82%	77%	75%	–	–	–
Time to assessment from symptom onset (months)^3^	10.2 ± 7.7	10.1 ± 8.0	10.2 ± 7.6	–	–	–	*0.97*
	7.4 [5.1, 14.3]	7.3 [5.1, 14.0]	7.5 [5.0, 15.0]				0.00
	0.5–36.0	0.6–35.0	0.5–36.0				
Time to death from study visit (months)^3^	6.9 ± 7.4	8.6 ± 7.6	6.5 ± 7.7	–	–	–	*0.60*
	3.6 [1.4, 9.8]	5.7 [2.9, 13.6]	3.5 [1.2, 8.9]				0.10
	0.4–38.3	0.5–29.5	0.4–38.3				
Time to death from symptom onset (months)^3^	17.1 ± 12.1	17.5 ± 12.8	16.7 ± 11.7	–	–	–	*0.75*
	15.5 [8.0, 23.7]	15.0 [8.5, 23.0]	16.2 [7.4, 23.9]				0.06
	1.1–64.5	2.1–64.5	1.1–56.8				
Barthel Index	67.8 ± 32.1	77.2 ± 28.9	59.0 ± 32.6	–	–	–	***0.004***
	80.0 [50.0, 95.0]	90.0 [55.0, 100.0]	70.0 [30.0, 85.0]				0.59
	0.0–100.0	0.0–100.0	0.0–100.0				
MMSE	14.7 ± 8.7	17.4 ± 7.3	11.7 ± 9.1	29.4 ± 0.8	***<0.0001***	***<0.0001***	***<0.0001***
	16.0 [7.0, 22.0]	18.0 [12.0, 24.0]	10.0 [3.5, 20.0]	30.0 [29.0, 30.0]	**2.47**	**2.90**	**0.69**
	0.0–30.0	2.0–30.0	0.0–29.0	27.0–30.0			

Abbreviations: Barthel Index (scored on a scale of 0–100, with 100 indicating functional independence); ES, Effect Size; MMSE, Mini Mental Status Exam^30^; sCJD, sporadic Creutzfeldt–Jakob disease.

Independent samples *t*‐test were used to compare sCJD participants and healthy controls, as well as within sCJD groups to determine *P‐*values for significance.

*P* < 0.05 (bolded).

^1^ For all groups, mean ± SD, median [IQR], and range are listed for all baseline demographic and clinical characteristics unless otherwise specified.

^2^ Participants were included if we were able to calculate at least 2 of 4 composite scores.

^3^ We could not confirm date of death for six participants. One participant was excluded as they were still alive during time of analysis.

^4^ Hedges’ *g* was used for effect sizes. 0.2 to <0.5 = small; 0.5 to <0.8 = medium; 0.8 to <1.3 = large; >1.3 = very large.

* *P*‐value for chi‐square significance test between sCJD and healthy controls.

A sample of 135 age‐matched healthy controls (HC) recruited from other studies at the UCSF Memory and Aging Center were included for normative neuropsychological data. Control participants were determined to be clinically normal based on a consensus conference that reviewed neurological examination, neuropsychological testing, and informant reports (Clinical Dementia Rating (CDR) = 0).

### Standard protocol for participants with neuropsychological assessment

#### Baseline neuropsychological measures

1. Memory composite: (a) California Verbal Learning Test‐Second Edition, Short Form (CVLT‐II‐SF)^34^ and (b) Modified Rey‐Osterrieth Recall (Benson figure).^35^, ^36^ Scores for total immediate recall (sum of nine words recalled over four learning trials), 10‐minute delayed recall, and recognition discriminability (d‐prime derived from hits and false‐positive errors) were used from the CVLT‐II‐SF, and the 10‐minute delayed recall score was used from the Benson figure.
2. Language composite: (a) 15‐item Boston Naming Test (aBNT),^37^ (b) 16‐item version of the Peabody Picture Vocabulary Test‐Revised (PPVT‐R),^38^ and (c) Semantic Fluency (animal naming).^35^ Total correct items for each task were used in the analyses.
3. Visuospatial composite: (a) Modified Benson Figure Copy,^35^ and (b) Number Location subtest from the Visual Object and Space Perception battery.^39^
4. Executive functioning composite: (a) Modified Trail Making Test B (logarithmic transformation of correct lines per minute),^35^ (b) Stroop Color–Word Interference Task,^40^ (c) Design Fluency (Condition 1: Filled Dots) from the Delis–Kaplan Executive Function System ,^41^ (d) Lexical Fluency (D‐words per minute),^35^ and (e) Digit Span Backwards (longest span).^42^

### Other clinical assessments

Most participants were also administered the Barthel Index (*n* = 47, 77%),^43^ a measure of functional severity commonly used in prion diseases.^44^, ^45^

### Statistical analyses

Missing neuropsychological/cognitive test data are not uncommon in sCJD.^11^ We did not feel that imputing the worst possible score was valid, as there were several possible reasons for missing data. For example, a patient might have been slow and could not complete all of the tests in the time allotted during the study visit; in other circumstances the subject could not complete the cognitive battery due to study time factors or scheduling constraints. We therefore created cognitive domain composite scores. For each neuropsychological task, we calculated z‐scores for the sCJD participants based on the HCs. Z‐scores within each cognitive domain were then averaged to create the four cognitive composite scores.^46^ Composite scores were based on available baseline data for each participant. To be able to include some participants who were missing some cognitive domain data, we only calculated a composite if the participant completed a minimum number of tests for that domain, similar to published methodology:^46^ memory composite required 2 of 4 measures; language composite required 2 of 3 measures; visuospatial composite required 2 of 2 measures; and executive functioning composite required 3 of 5 measures. The calculated composite scores were then averaged to create a global composite z‐score for each sCJD participant. We assessed the proportion of participants who were impaired on each cognitive measure and composite, based on a z‐score ≤ −1.5. To account for unequal sample sizes, we calculated effect sizes using Hedges’ *g* to compare performance on each measure or composite, controlling for education, between sCJD and healthy controls.^47^ Similar to Cohen’s *d*, magnitude of effect sizes was interpreted using standardized categorization (0.2 to <0.5 = small; 0.5 to <0.8 = medium; 0.8 to <1.3 = large; >1.3 = very large).^48^, ^49^, ^50^ We report the effect size, rather than just the statistical significance, because it emphasizes the size of the difference and is less confounded by sample size.^51^

Survival times (“survival”) were calculated both from participants’ onset of first symptom and from UCSF baseline study visit until death. One patient alive at the time of analysis was censored at that date. Cox proportional hazard models were used to predict survival time. We tested the association of MMSE and survival time in the entire sample. For the neuropsychological survival analyses, we required participants to have at least two calculated cognitive composites (*n* = 61). Survival analyses were first performed without covariates. Based on a priori knowledge and results from this study, we also ran a separate model controlling for codon 129 polymorphism and disease severity (Barthel Index). sCJD molecular classification was not included in the models because of low case numbers for certain subtypes (Supplemental Table S1). Statistical analyses were performed using STATA version 14.2 (Statacorp, College Station, TX).

## Results

### Baseline demographic and clinical characteristics of sample

Demographic and clinical characteristics are reported in Table 1 and Supplemental Table S1.

### Neuropsychological test performance

Raw and normative scores for baseline neuropsychological measures and cognitive composite scores are shown for sCJD participants (Table 2). For composites, the highest effect size was for the executive composite (*P* < 0.0001, Hedges’ *g* = 3.73), although the memory composite effect size was also quite high (*P* < 0.0001, Hedges’ *g* = 3.16). The highest effect sizes for single tests were observed for measures of verbal memory delayed recall (*P* < 0.0001, Hedges’ *g* = 4.08) and Stroop Inhibition (*P* < 0.0001, Hedges’ *g* = 3.14). Very large effect sizes (>1.3) were seen across most tasks, however, including modified trails (*P* < 0.0001, Hedges’ *g* = 2.94), animal naming (*P* < 0.0001, Hedges’ *g* = 2.90), visuospatial memory (*P* < 0.0001, Hedges’ *g* = 2.64), verbal learning (*P* < 0.0001, Hedges’ *g* = 2.28), lexical fluency (*P* < 0.0001, Hedges’ *g* = 2.24), design fluency (*P* < 0.0001, Hedges’ *g* = 2.11), confrontation naming (*P* < 0.0001, Hedges’ *g* = 2.04), Digit Span Backwards (*P* < 0.0001, Hedges’ *g* = 2.00), and verbal recognition discriminability (d‐prime; *P* < 0.0001, Hedges’ *g* = 1.32). Large effect sizes (>0.8, ≤1.3) were seen for PPVT‐R (*P* < 0.0001, Hedges’ *g* = 1.19), visuospatial copy (*P* < 0.0001, Hedges’ *g* = 1.15), and number location (*P* < 0.0001, Hedges’ *g* = 1.09). The vast majority of sCJD participants were impaired on memory (95.5%) and executive functioning (86.7%) composite scores. Within the memory domain, CVLT‐II delayed recall (verbal memory) was the most commonly impaired, followed by CVLT‐II immediate recall, Benson figure delayed recall, and CVLT‐II recognition discriminability (d‐prime).

**Table 2:** Baseline neuropsychological data in sCJD.^1^

	*N*	Raw scores M (SD)	*P*‐value^1^	Effect size (Hedges’ *g*)^1^, ^2^	z‐scores M (SD)^1^	% Impaired
Memory						
CVLT‐II‐SF: immediate recall	49	15.73 (7.62)	<0.0001	2.28	−4.44 (2.35)	87.8%
CVLT‐II‐SF: delayed recall	49	1.55 (1.95)	<0.0001	4.08	−4.67 (1.38)	97.7.%
CVLT‐II‐SF: recognition discriminability (d‐prime)	43	1.48 (1.20)	0.003	1.32	−6.06 (3.88)	86.0%
Benson figure: delayed recall	47	4.00 (3.95)	<0.0001	2.64	−3.68 (1.68)	87.2%
Memory composite^3^	49	‐	<0.0001	3.16	−4.88 (1.96)	95.5%
Language						
aBNT	51	10.78 (3.72)	<0.0001	2.04	−2.28 (2.71)	48.1%
PPVT‐R	44	12.93 (4.01	<0.0001	1.19	−2.10 (3.58)	45.5%
Animal naming	52	6.88 (4.64)	<0.0001	2.90	−3.50 (0.97)	98.1%
Language composite^3^	51	–	<0.0001	2.46	−2.67 (2.17)	74.5%
Visuospatial						
Benson figure: copy	51	10.94 (5.84)	<0.0001	1.15	−4.23 (5.34)	62.7%
VOSP	37	6.57 (3.01)	<0.0001	1.09	−2.32 (2.57)	52.6%
Spatial composite^3^	49	−	<0.0001	1.29	−4.00 (4.69)	65.3%
Executive functioning						
Modified trails^4^	39	1.62 (1.11)	<0.0001	2.94	−4.21 (2.47)	82.5%
Stroop inhibition	35	19.91 (12.33)	<0.0001	3.14	−3.39 (1.15)	91.7%
Design fluency (Condition 1)	38	4.26 (3.02)	<0.0001	2.11	−1.96 (0.96)	76.9%
Lexical fluency (D words)	53	5.04 (4.46)	<0.0001	2.24	−2.74 (1.27)	85.2%
Digit span backwards	49	2.29 (1.46)	<0.0001	2.00	−2.33 (1.10)	85.7%
Executive composite^3^	45	–	<0.0001	3.73	−2.92 (1.08)	86.7%

Independent samples *t*‐test were used to compare sCJD participants and healthy controls to determine *P‐*values for significance and Hedges’ *g* for effect sizes.

Abbreviations: California Verbal Learning Test‐Second Edition, Short Form (CVLT‐II‐SF)^33^; Modified Rey‐Osterrieth Copy and Recall (Benson figure)^34^, ^35^; 15‐item abbreviated Boston Naming Test (aBNT)^36^; 16‐item abbreviated version of the Peabody Picture Vocabulary Test‐Revised (PPVT‐R)^37^; Semantic Fluency (animal naming)^34^; Number Location subtest from the Visual Object and Space Perception (VOSP) battery^38^; Modified Trail Making Test B (modified Trails)^34^; Stroop Color‐Word Interference Task (Stroop Inhibition)^39^; Design Fluency (Condition 1: Filled Dots) from the Delis–Kaplan Executive Function System (D‐KEFS)^40^; Lexical Fluency (D words per minute)^34^; Digit Span Backwards.^41^ Means (M); sCJD, sporadic Creutzfeldt‐Jakob disease; standard deviation (SD).

^1^ Compared to age and education matched controls.

^2^ Effect size for Hedges’ *g* considered large if >0.8 and very large if >1.3 (see text).

^3^ Cognitive composite scores are averaged *z*‐scores. See Methods section for a detailed description of the procedure for calculating cognitive composites.

^4^ Log‐transformed score to normalize data; denotes correct number of lines per minute.

### Survival predictions

We first assessed age, sex, Barthel Index and codon 129 as predictors of survival time. When age and sex were entered together, neither was significantly associated with survival (age: HR = 1.00, 95% CI [0.98, 1.02], *P* = 0.81; sex: HR = 0.90, 95% CI [0.62, 1.31], *P* = 0.59). In a univariate model, the Barthel Index (HR = 0.98, 95% CI [0.98, 0.99], *P* < 0.0001) was a statistically significant predictor of survival. Heterozygosity in codon 129 tended to be associated with longer survival, although this did not quite reach statistical significance (HR = 1.25, 95% CI [0.98, 1.61], *P* < 0.06).

**Figure 1:**
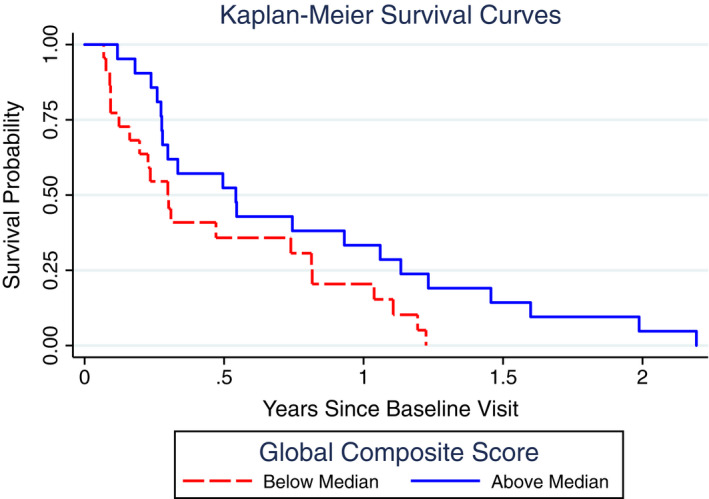
Kaplan–Meier survival curves for global composite score. Kaplan–Meier curves showing the probability of survival in Creutzfeldt–Jakob disease as a function of the median split of the global cognitive composite score (average z‐scores of four cognitive domain composite scores). Lower scores (dashed red line) predicted shorter survival time.

**Figure 2:**
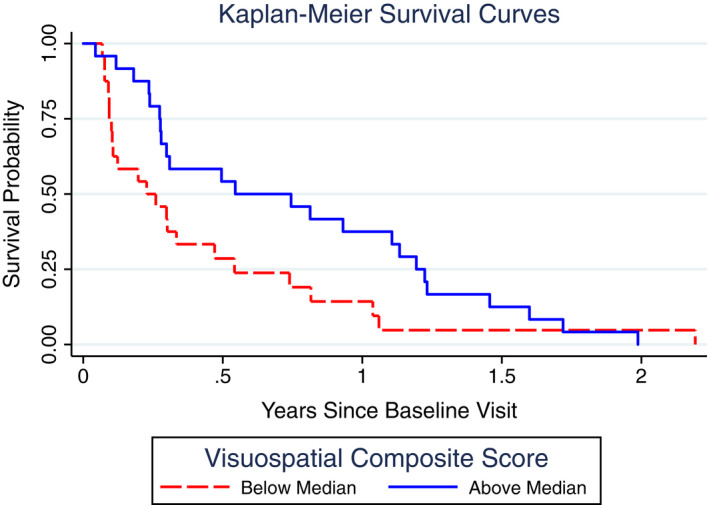
Kaplan–Meier survival curves for visuospatial composite score. Kaplan–Meier curves showing the probability of survival in Creutzfeldt–Jakob disease as a function of the median split of the visuospatial cognitive composite score (average z‐score of visuospatial task scores). Lower scores (red dashed line) predicted shorter survival time.

Results of the neuropsychological survival analysis found that MMSE performance was not significantly associated with survival (HR = 0.99, 95% CI [0.97, 1.01], *P* = 0.37) (Table 3). Worse performance on the global cognitive composite, however, predicted shorter survival time (HR = 0.65, 95% CI [0.51, 0.83], *P* < 0.0001; Figure 1). In our analysis of the domain‐specific cognitive composites, lower visuospatial (HR = 0.82, 95% CI [0.75, 0.89], *P* < 0.0001; Figure 2) and memory (HR = 0.82, 95% CI [0.69, 0.96], *P* = 0.01) scores were associated with shorter survival. Importantly, when including all four composite scores in the same model, the visuospatial composite score was the only statistically significant predictor (Visuospatial: HR = 0.82, 95% CI [0.73, 0.94], *P* = 0.003; Memory: HR = 0.87, 95% CI [0.68, 1.10], *P* = 0.25; Language: HR = 0.87, 95% CI [0.58, 1.32], *P* = 0.52; Executive: HR = 1.01, 95% CI [0.62, 1.67], *P* = 0.96). The hazard ratio for the global composite (HR = 0.65) can be interpreted as a 35% increased risk of mortality (1.00‐0.65 = 35%) for each 1 SD worsening of global composite performance. Similarly, the HR for visuospatial composite (HR = 0.82) can be interpreted as an 18% increased risk of mortality (1.00‐0.82 = 18%) for each 1 SD worsening of visuospatial performance. Table 4 shows how the hazard function can also be used to derive estimates of survival to different time points based on different levels of cognitive composite of interest. For example, based on the survival function, we estimate that a patient with a visuospatial score of −5 (i.e., 5 SDs below the mean) only has a 29% probability of surviving for 6 months (95% CI [16, 44]), whereas the probability of a patient with a visuospatial score of 0 (i.e., average) surviving to 6 months is 64% (95% CI [46, 77]). In Table 4, we note important caveats when trying to use this data to predict survival in a patient with sCJD.

**Table 3:** Survival time hazard ratios for MMSE and cognitive composites with and without various covariates.

Cognitive test or composite	Without covariates				With Barthel index and Codon 129 covariates			
	*N* (died)**	HR	*P*	95% CI	*N* (died)**	HR	*P*	95% CI
MMSE	118 (117)	0.99	0.373	0.97, 1.01	98 (97)	1.01	0.648	0.98, 1.03
Global^1^	**43 (42)**	**0.65**	**<0.0001** *	**0.51, 0.83**	**33 (32)**	**0.68**	**0.026** *	**0.48, 0.95**
Visuospatial^2^	**48 (47)**	**0.82**	**<0.0001** *	**0.75, 0.89**	**36 (35)**	**0.82**	**0.001** *	**0.72, 0.92**
Memory^2^	**46 (46)**	**0.82**	**0.014** *	**0.69, 0.96**	35 (35)	0.88	0.269	0.71, 1.10
Language^2^	49 (48)	1.00	0.946	0.89, 1.14	38 (37)	1.00	0.982	0.80, 1.25
Executive^2^	44 (43)	0.96	0.833	0.68, 1.36	33 (32)	1.03	0.921	0.63, 1.68

MMSE, Mini Mental Status Exam.^30^

^1^ Global composite score is the average of four cognitive domain composite scores. To explain the hazard ratio (HR) in this table, the HR for the global composite (HR = 0.65) can be interpreted as a 35% increased risk of mortality (1.00‐0.65 = 35%) for each 1 SD worsening of Global composite performance. See Methods section for full description of how the cognitive composites were developed.

^2^ Cognitive domain composite scores are averaged z‐scores.

* *P* < 0.05 (bolded).

** One patient alive at the time of analysis was censored using Cox proportional hazard models.

**Table 4:** Probability of survival from testing to 3, 6, and 12 months for different visuospatial and global cognition Z‐scores.^1^, ^2^

Cognitive composite z‐score	3 months %^3^ [95% CI]	6 months %^3^ [95% CI]	1 2 months %^3^ [95% CI]
Visuospatial^4^			
0	81.6 [66.9, 90.2]	63.7 [45.9, 77.0]	41.6 [24.9, 57.5]
−2.5	71.4 [55.6, 82.4]	47.3 [31.6, 61.5]	23.3 [11.6, 37.2]
−5	57.1 [40.2, 70.8]	28.8 [15.5, 43.6]	8 [2.4, 20.5]
Global^5^			
0	90.8 [76.5, 96.7]	80.2 [58.4, 91.3]	66.5 [39.7, 83.5]
−2.5	75.5 [59.8, 85.8]	52.6 [35.9, 66.8]	30.4 [16.7, 45.3]
−5	44.1 [22.8, 63.6]	15.3 [3.8, 34.1]	3.1 [0.2, 14.3]

^1^ Only cognitive composites with significant hazard ratios (visuospatial and global) were used for this analysis.

^2^ Important caveats: we must recommend caution when interpreting such a table, as these data apply to a cohort with detailed cognitive assessment and whom were on average 60% of their way through their disease course; Furthermore, although large for a CJD cohort, this is still a cohort that has its own referral biases. Ideally, this type of analysis should be conducted within an even larger cohort and these predictions should be validated in an independent sample. Lastly, these cognitive scores were derived by calculating composites on a specific set of cognitive assessments.

^3^ The same survival model used to predict the hazard ratio (see Methods) also was used to derive the probability of survival to these three time points.

^4^ Cognitive composite scores are averaged z‐scores. Note that a z‐score of 0 means the score is equivalent to the average for healthy controls (HC) and ‐5 means a score that is 5 standard deviations below the average for HC.

^5^ Global composite score is the average of four cognitive domain composite scores.

In subsequent analyses, we assessed whether domain‐specific results were independent of other known predictors of survival (i.e., codon 129, Barthel Index). The visuospatial composite score remained a statistically significant independent predictor when controlling for the Barthel Index and codon 129 (HR = 0.82, 95% CI [0.72, 0.92], *P* = 0.00). Similar results were observed for the global composite score (HR = 0.68, 95% CI [0.48, 0.95], *P* = 0.03), with the Barthel Index also independently being a significant predictor within the same model (HR = 0.97, 95% CI [0.96, 0.99], *P* = 0.01).

## Discussion

The present study sought to characterize baseline neuropsychological profiles in sCJD participants and determine whether baseline cognition is associated with survival time in this cohort. To our knowledge, this is the largest sCJD cohort with neuropsychological data published to date. Participants were on average 60% into their total disease course at time of assessment. Our standardized baseline neuropsychological data revealed global cognitive compromise at the group level across domains of memory, language, visuospatial functioning, and executive functioning, expanding the existing literature predominantly focused on cognitive impairments in mixed prion cohorts, case reports, or variant and other prion disease subtypes.^7^, ^8^, ^12^ The most frequent impairments were in the domains of executive functioning and memory, consistent with prior literature, indicating that these domains are commonly affected in prion disease.^7^, ^9^, ^12^ Furthermore, measures of verbal and visuospatial memory and executive functioning demonstrated the largest effect sizes between sCJD participants and healthy controls.

More importantly, our results suggest that baseline cognitive testing is a prognostic indicator of survival time in sCJD, even when accounting for two other measures that independently are associated with survival: *PRNP* codon 129 polymorphism and Barthel Index. Although greater baseline cognitive impairment in global cognition, memory, and visuospatial domains each predicted shorter survival time when modeled separately, visuospatial impairment remained a significant predictor when all other cognitive domain scores were entered into the same model. Furthermore, visuospatial performance remained a significant predictor even when controlling for other known predictors, such as functional impairment (i.e., Barthel Index) and *PRNP* codon 129 polymorphism. Consistent with this finding, the Heidenhain variant of sCJD, characterized by early visual disturbances and oculomotor dysfunction,^28^, ^52^ has been associated with more rapid disease progression.^28^ Although it is unknown why this variant is associated with such rapid disease course, it might be related to molecular subtype; the MM1 molecular subtype, associated with the shortest survival times,^17^, ^53^, ^54^ is the most common in the Heidenhain variant, although a recent study has also shown associations with the MM2‐C prion typing.^27^ In our sample, however, those with visuospatial impairment showed a wide range of molecular subtypes (data not shown), suggesting that these findings are not simply driven by molecular subtype.

Our findings should be interpreted in light of several limitations. First, it is important to note that these results apply to a subsample – about one half – of our sCJD cohort who could participate in the MMSE with or without more comprehensive neuropsychological assessment. This highlights the importance of complementary predictors of survival – such as functional scales, CSF, blood or imaging biomarkers – that might aid in prognosis regardless of a person’s ability to participate in cognitive testing. Thus, replication of results in a larger independent sample is warranted. Second, our cohort had a somewhat higher proportion of codon 129 VV and MV cases, and a lower proportion of MM cases, than some other Western cohort studies,^17^, ^54^ but we still had representation of all molecular subtypes. A larger analysis with sufficient numbers of all molecular subtypes is needed to include this variable in the model. Third, the inclusion of subjects who were able to complete neuropsychological testing might have introduced a survivor bias. Therefore these results should be generalized only to sCJD patients who are able to participate in MMSE testing. Fourth, using composite scores to compensate for missing data might not have adequately captured instances when a patient could not perform a task due to severe impairment in that domain. Thus, some domains might be even more impaired in general in sCJD than shown in our study. Alternatively, impairment on a cognitive task might be due to impairment in a different cognitive domain than that being assessed. For example, a patient without visuospatial impairment might not be able to perform a visuospatial task due to general confusion, aphasia, or other issues.

We also recommend caution when using Table 4 to help predict a patient’s survival for three reasons: (1) these data apply to those in our cohort who had detailed cognitive assessment, and such a assessment might not be done in standard clinical practice; (2) the scores were derived by calculating composites on a specific set of cognitive assessments; and (3) sCJD subjects were on average 60% of the way through their disease course. Although our cohort was large for a CJD cohort, it still has its own biases. We therefore recommend that future analyses be conducted in an even larger cohort and that these predictions be validated in an independent sample.

A key strength of our study, however, is that the battery used for this study was designed to be administered to participants with cognitive impairments, takes about 1 hour to administer, and features shortened versions of many common neuropsychological tests, reducing patient burden.^35^ Another strength is that we used the modified Rey‐Osterrieth (Benson) figure to evaluate visuoconstruction performance. The Benson figure has fewer elements than the standard Rey‐Osterrieth figure and therefore might be more efficiently administered to impaired participants. Interestingly, visuospatial functioning was one of the least frequently impaired domains, present in about two‐thirds of subjects; the variability in performance among participants might underlie its utility in predicting survival. Interestingly, the MMSE was not a significant predictor of survival time in this sample. Future studies might also benefit from utilizing tablet‐based cognitive screens, such as the UCSF Brain Health Assessment, which might be more sensitive to cognitive deficits and weight visuospatial performance to a greater degree than the MMSE.^55^

This work advances current knowledge of neuropsychological performance in sCJD and provides initial evidence that cognitive testing could be a noninvasive prognostic tool. Historically, prognostication and markers of survival time in sCJD have been limited to demographics^18^, fluid biomarkers,^17^, ^20^, ^22^, ^23^, ^24^, ^25^, ^54^ codon 129,^18^, ^19^, ^20^, ^21^ and molecular classification,^17^, ^54^ some of which have had limited utility and are often invasive and expensive. Recently, we showed that blood‐based biomarkers of neurodegeneration also have promise for predicting survival time in sCJD.^20^ Neuropsychological testing could potentially be utilized in conjunction with blood‐based biomarkers and other noninvasive, lower‐cost strategies to predict survival time. Accurate prognostication provides valuable information for clinical trials seeking to stratify participants based on expected trajectories. Lastly, until a cure for sCJD is found, knowledge of an estimated time until death is important information that would help the treatment team assist patients and families with future planning.

## Conflicts of Interest

MDG receives research support from the NIH/NIA (grant R01 AG031189, R56AG055619, R01AG062562) and the Michael J. Homer Family Fund. He has consulted for Quest Diagnostics, 3D Communications, Adept Field Consulting, Advanced Medical Inc., Best Doctors Inc., Grand Rounds Inc., Second Opinion Inc., Gerson Lehrman Group Inc., Guidepoint Global LLC, MEDACorp, Market Plus, LCN Consulting, Optio Biopharma Solutions, Teva Pharmaceuticals, Trinity Partners LLC, Biohaven Pharmaceuticals, Quest Diagnostics and various medical‐legal consulting. He has received speaking honoraria for various medical center lectures and from Oakstone publishing. He has received past research support from Alliance Biosecure, CurePSP, the Tau Consortium, Quest Diagnostics, and NIH. He serves on the board of directors of San Francisco Bay Area Physicians for Social Responsibility and on the editorial board of *Dementia & Neuropsychologia*. JHK receives research support from NIH, the Tau Consortium, and the Larry H. Hillblom Foundation and provides consultation to Biogen. HJR has received research support from Biogen Pharmaceuticals, has consulting agreements with Wave Neuroscience and Ionis Pharmaceuticals, and receives research support from NIH. The rest of the authors have nothing to report.

## Author Contributions

(1) SES, AMS, NCW, KBC, HJR, JHK, and MDG were involved in study conception and design.

(2) SES, AMS, NCW, MC, AG, SM, KO, KW, KB, SF, MGC, IEA, and MDG were involved in data acquisition and analysis.

(3) SES, AMS, NCW, KBC, and MDG were involved in drafting of the manuscript and figures.

## Supporting information


**Table S1.** Codon 129 and molecular classification characteristics of the sCJD cohort.
