# Supplementary material for: Baseline neuropsychological profiles in prion disease predict survival time
Source: Ann Clin Transl Neurol. 2020 Sep 9;7(9):1535–45. doi: 10.1002/acn3.51115 (PMC7480924; doi:10.1002/acn3.51115)
Supplement: Supplementary file 1 — Table S1. Codon 129 and molecular classification characteristics of the sCJD cohort. [file ACN3-7-1535-s001.docx]

Supplementary Table 1. Codon 129 Characteristics

|  | **sCJD Total Sample** | **Neuropsychological Survival Analyses** | **sCJD MMSE-Only Sample** |
| --- | --- | --- | --- |
| **N** | 118 | 61 | 57 |
| **Total pathologically**  **confirmed** | 90 (77) | 42 (69) | 48 (86) |
| **Total probable** | 28 (23) | 19 (31) | 9 (14) |
| ***PRNP* codon 129 Available**  **N (%)** | 114 (97%) | 59 (97%) | 55 (96%) |
| **Molecular Classification Typing**  **Available**  **N (%)** | 81 (69%) | 35 (57%) | 46 (81%) |
| **MM^a^** | 40 (34) | 15 (25) | 25 (44) |
| **MM1** | 9 (7) | 3 (5) | 6 (10) |
| **MM2** | 16 (14) | 5 (8) | 11 (20) |
| **MM 1+2** | 8 (7) | 2 (4) | 6 (10) |
| **MM?** | 7 (6) | 5 (8) | 2 (4) |
| **MV** | 52 (44) | 33 (54) | 19 (33) |
| **MV1** | 10 (8) | 4 (7) | 6 (10) |
| **MV2** | 14 (12) | 8 (13) | 6 (10) |
| **MV 1+2** | 10 (8) | 6 (10) | 4 (7) |
| **MV?** | 18 (16) | 15 (24) | 3 (6) |
| **VV** | 22 (19) | 11 (18) | 11 (19) |
| **VV1** | 1 (1) | 1 (1) | 0 (0) |
| **VV2** | 10 (8) | 4 (7) | 6 (10) |
| **VV 1+2** | 3 (3) | 2 (3) | 1 (2) |
| **VV?** | 8 (7) | 4 (7) | 4 (7) |

^a^ methionine (M) or valine (V) alleles in codon 129 polymorphisms.

Abbreviations: sCJD=sporadic Creutzfeldt-Jakob disease; MMSE=Mini Mental Status Exam.^30^
